# Peer Review of “Detecting Substance Use Disorder Using Social Media Data and the Dark Web: Time- and Knowledge-Aware Study”

**DOI:** 10.2196/58320

**Published:** 2024-05-01

**Authors:** Anonymous

**Keywords:** opioid, substance use, substance use disorder, social media, US, opioid crisis, mental health, substance misuse, crypto, dark web, users, user perception, fentanyl, synthetic opioids, United States

*This is the peer-review report for “Detecting Substance Use Disorder Using Social Media Data and the Dark Web: Time- and Knowledge-Aware Study.”*

## Round 1 Review

This study [1] can be considered for publication if the researchers are able to revise it to improve the clarity of the objective, theoretical relevance, and practical value. I will suggest that there should be a segment on the objective of the study immediately after the introduction. This will help in giving the study a direction. Please, include this citation:

Obosi AC, Fatunbi AM, Oyinloye O. Peer pressure and substance use as predictors of mental health among in-school adolescents in Nigeria. *Ianna J Interdisciplinary Stud*. 2022;4(1):1‐9.

Explain the implication of your findings to other countries, that is, give the study an international outlook.
